# Integrating Lifestyle Medicine and the Geriatric 5Ms for Sustained Behavior Change in Older Adults

**DOI:** 10.1093/geroni/igaf122.2111

**Published:** 2025-12-31

**Authors:** Rebecca Lassell, Brian Andonian

## Abstract

This symposium introduces a new framework of Lifestyle Medicine (LM) that acknowledge the complex contexts and care needs of older adults to promote and sustain behavior change. The framework of Lifestyle Medicine for Older Adults integrates their lived experiences (e.g., social determinants of health, built environment), the 5M framework of geriatric medicine (i.e., Mind, Mobility, Medications, Multicomplexity, Matters Most) and the six pillars of LM (i.e., nutrition/diet, physical activity/exercise, restorative sleep, stress management, avoidance of risky substances, positive social connections) through a novel transdisciplinary approach. Guided by the framework, we will describe: 1) lifestyle recommendations for older adults: challenges and opportunities for practice, research, and policy; 2) how applying the framework using a transdisciplinary approach might address these challenges, and 3) recommendations for prescribing lifestyle for older adults tailored to their daily contexts and experiences. Five presenters will provide clinical and research examples that embody the framework. Presentations will range from the current evidence for sustained behavior change in older adults, different methodological approaches for LM analyses in publicly available data, observational studies, and community-based interventions.

